# Breast cancer-derived CAV1 promotes lung metastasis by regulating integrin α6β4 and the recruitment and polarization of tumor-associated neutrophils

**DOI:** 10.7150/ijbs.94153

**Published:** 2024-10-14

**Authors:** Qing Lin, Siwen Zong, Yi Wang, Youjia Zhou, Keqin Wang, Fuxiu Shi, Jiayang Wang, Mingrui Feng, Wenting Luo, Lifang Zhang, Hui Lin, Lixia Xiong

**Affiliations:** 1 The MOE Basic Research and Innovation Center for the Targeted Therapeutics of Solid Tumors, School of Basic Medical Sciences, Jiangxi Medical College, Nanchang University, Nanchang 330006, China.; 2 Second Clinical Medical College, Nanchang University, Nanchang 330006, China.; 3 First Clinical Medical College, Nanchang University, Nanchang 330006, China.; 4 Key Laboratory of Functional and Clinical Translational Medicine, Xiamen Medical College, Fujian Province University, Xiamen 361023, China.

**Keywords:** Caveolin-1, breast cancer, lung metastasis, neutrophils, PMN

## Abstract

Lung metastasis in breast cancer (BC) patients is one of the main reasons for their high mortality rate. The most prevalent BC small extracellular vesicles (sEVs receptor, integrin α6β4, has been found to interact with surfactant-associated protein (SFTPC) in lung epithelial cells, making BC more likely to metastasize to the lung. Tumor-associated neutrophils (TANs) play an essential role in BC lung metastasis as a component of the lung pre-metastatic niche (PMN) with two sides. It has been demonstrated that Toll-like Receptor4 (TLR4) can participate in signaling, such as NF-B and NLRP3, to facilitate tumor metastasis. A cellular membrane structural protein called caveolin-1 (CAV1) is associated with BC's proliferation, metastasis, and immunological control. According to our previous research, CAV1 on BC-derived sEVs facilitates the formation of the lung PMN by enhancing tenascin-C (TnC) secretion in lung fibroblasts to promote the deposition of ECM, by increasing the expression of PMN marker genes and inflammatory chemokines in lung epithelial cells, and by supporting N2-type polarization of lung macrophages via inhibiting the PTEN/CCL2/VEGF-A axis. More research is needed to determine how sEVs-mediated CAV1 facilitates BC-targeted metastasis to the lungs. By creating a stable-translocating cell line that stably interfered with CAV1 and a mouse model of BC lung metastasis, we investigated how sEVs-mediated CAV1 promotes BC lung metastasis and TAN recruitment and polarization *in vivo* and *in vitro*. In this study, we showed that CAV1 increases the likelihood that BC lung metastasis would occur by controlling the expression of integrin α6β4 and via boosting TANs recruitment and polarization through activating the TLR4-NF-B-IL-6/CCL2 and TLR4/NF-B/NLRP3 signaling pathways. According to our findings, CAV1 regulates integrin α6β4 and modulates TLR4 signaling, both of which are critical for BC lung metastasis. This finding may open new avenues for BC lung metastasis prevention and treatment.

## Introduction

The latest global cancer data in 2023 showed that breast cancer (BC) has the first incidence rate and the second mortality rate among all female patients 1. Lung metastasis is a widespread distant BC metastasis, and the average survival of patients with lung metastasis is only 25 months 2, which deserves focused attention.

Prior to the initiation of tumor metastasis, the primary tumor secretes a large amount of tumor-secreted factors to the secondary site, which creates a favorable microenvironment for the subsequent metastasis, i.e., Pre-metastatic niche (PMN), which can make the distant organs suitable for colonization by circulating tumor cells 3. Primary tumor-derived components, tumor-mobilized bone marrow-derived cells (BMDCs), and the local stromal microenvironment of host (or future metastatic organ components) are three key factors in the formation of PMN 4. Lung epithelial cells, as the most widespread stromal cells in the lungs, are capable of malignant proliferation, Epithelial-mesenchymal transition (EMT), or crosstalk with extracellular matrix in response to various tumor stimuli 5. In contrast, neutrophils, as a type of BMDC, are known as tumor-associated neutrophils (TANs) in cancer. TANs in PMN have the potential to polarize into anti-tumor tumor-associated neutrophils (N1 TAN) or pro-tumor tumor-associated neutrophils (N2 TANs) in response to tumor-secreting factors 6. Therefore, investigating the mechanism of how tumor-secreted factors lead to these changes in lung tissue cells is essential for combating BC lung metastasis and finding new effective therapeutic targets.

As an extracellular vesicle with a diameter of 30-150 nm, small extracellular vesicles (sEVs) can be secreted by a wide range of cells, delivering a variety of signaling molecules, such as proteins and nucleic acids, and regulating intercellular communication under normal and pathological conditions 7. Biomolecules encapsulated by sEVs have more stable properties and better biocompatibility than free biomolecules 8. SEVs produced by cancer cells can not only play an autocrine role in the local microenvironment of the tumor but also play a paracrine role. For example, they can orchestrate the primary tumor microenvironment and promote the proliferation, invasion, and migration of tumor cells 9, 10. They can also pass through the blood circulation to distant target organs, thus exerting an endocrine effect and inducing the production of PMN in the target organs, which is conducive to distant metastasis of cancer 11.

Caveolin-1 (CAV1) is a functional protein of cytoplasmic membrane microvesicles, which forms homodimers consisting of multiple monomers through N-terminal sequence interactions and interacts with the conserved Caveolin binding domain (CBD) within numerous signaling proteins through the N-terminal Caveolin scaffolding domain (CSD) function through interaction. CAV1 also plays a vital role in the progression of various diseases, including cancer, through specific amino acid phosphorylation and regulation of other molecular expressions. CAV1 promotes metastasis and multi-drug resistance in late-stage cancer. CAV1 is more highly expressed in advanced cancer than benign tissues and is associated with poor prognosis. Overexpression of CAV1 is positively associated with increased cell survival, anchorage-dependent growth, EMT, migration, invasion, and resistance to antitumor drugs 12, 13. It has been shown that phosphorylation of CAV1 on tyrosine at position 14 (CAV1-pY14) is another essential requirement for enhancing the metastatic potential of cancer cells *in vitro* and *in vivo* in addition to the expression of CAV1 at the plasma membrane 14. CAV1 acts intracellularly and can be encapsulated in sEVs and transported outside the cell to perform its functions 15. In addition, CAV1 can be involved in regulating the expression of proteins on sEVs. Some results of mass spectrometry analysis showed that in the presence of CAV1, BC cells recruited and secreted sEVs containing cell adhesion-associated proteins, which could significantly promote tumor migration and invasion 16. CAV1 centrally regulates sEVs biogenesis and cargo sorting of exosomal proteins by controlling cholesterol content in endosomal compartments/multi-vacuolar bodies 17. All these suggest that CAV1 can regulate the assembly of cargoes on sEVs, simultaneously, and can be loaded on sEVs for translocation to distant organs to influence tumor progression. The results of our previous study showed that sEVs from BC function as a signaling molecule to mediate intercellular communication and regulate the PMN prior to lung metastasis by controlling the expression of PMN marker genes and inflammatory chemokines in lung epithelial cells, encouraging the release of tenascin-C (TnC) in lung fibroblasts to cause the deposition of extracellular matrix (ECM), and inhibiting the PTEN/CCL2/VEGF-A signaling pathway in lung macrophages to facilitate their M2-type polarization and angiogenesis 18.

Integrins (ITGs) are essential cell surface receptor members, a family of twenty-four transmembrane heterodimers consisting of eighteen α-subunits (ITGAs) and eight β-subunits (ITGBs), which mainly mediate cell-to-cell or cell-to-extracellular matrix adhesion 19, which act as membrane molecules to mediate signaling through unique signaling pathways and participate in the recognition, stretching and migration of cells from almost every step from cancer primary to metastasis 20. Integrins are involved in proliferative signaling, tumor invasion and metastasis, apoptosis evasion, and angiogenesis stimulation by activating downstream molecules such as PI3K, AKT, and MAPK 21-23. CAV1, a membrane molecule linked to cancer metastasis, participates in the integrin signaling pathway. CAV1-pY14 is regulated by Focal adhesion kinase (FAK) in response to the stimulation of changes in cellular adhesion, and integrin-mediated adhesion induces the autophosphorylation and activation of FAK-Tyr397, which recruits Src; the Src family of kinases is responsible for CAV1- pY14, and additionally, phosphorylated CAV1 specifically contributes to Src recruitment to the plasma membrane, leading to Src aggregation and activation at focal adhesions 24. It was shown that the expression of integrins β1, β3, and αv was significantly downregulated after stable transfection of shRNA-CAV1, which successfully inhibited the migration of lung cancer cells 25. The above studies suggest that the signaling crosstalk between CAV1 and integrins plays a vital role in tumor development and metastasis.

In addition, integrins, as the most highly expressed receptors on the surface of sEVs, have been shown to function in directing sEVs to specific tissue sites, with a propensity to support metastasis of different types of cancer to specific organs 26. Lung-friendly cancer cells secrete sEVs enriched in integrin α6β4, and sEVs derived from lung-ophilic BC cells preferentially fuse with lung fibroblasts and lung epithelial cells; integrin α6β4 may interact with extracellular matrix components to mediate sEVs uptake at specific target sites within the lung, which in turn activates Src phosphorylation 27-29. Surfactant-associated protein (SFTPC) is a receptor protein on the surface of alveolar epithelial cells. Integrin α6β4 on sEVs can interact with SFTPC on the surface of lung epithelial cells, thereby enabling breast cancer-targeted translocation to the lungs 26. Integrin α6β4 on the surface of BC cells can recognize SFTPC with lung epithelial cells, further activating the downstream PI3K/AKT signaling pathway and the phosphorylation of Src to promote the malignant proliferation and invasion of cells 30. These suggest that either integrin α6β4 on the surface of cancer cells or tumor cell-derived sEVs can interact with SFTPC to promote lung metastasis.

Toll-like Receptor4 (TLR4) plays an essential role in the immune surveillance of tumor cells, and it has been shown that CAV1 is involved in the regulation of TLR4^31^-^33^. TLR4 is also involved in various signaling pathways, such as Nuclear factor-kappaB (NF-κB), NOD-like receptor thermal protein domain associated protein 3 (NLRP3), etc. NF-κB is one of the significant factors linking cancer and inflammation, and it induces cytokines of the immune response, including TNFα, IL-1, IL-6, and IL-8, as well as adhesion molecules therein, which allow inflammatory sites to leukocytes can be recruited. Cancer progression is often accompanied by elevated expression of Matrix metalloproteinases (MMPs), which release extracellular matrix and promote tumor invasive migration. In addition, NF-κB can regulate vascularization at the tumor site by elevating Vascular endothelial growth factor (VEGF) and its receptors, accelerating tumor progression 34. All the above studies suggest that CAV1 may affect NF-κB downstream signaling via regulating TLR4, thereby influencing the phenotypic transformation of neutrophils in PMN.

Currently, there are no relevant studies on whether sEVs-mediated CAV1 affects BC transfer to the lungs by regulating integrin α6β4 expression and the effects and mechanisms of sEVs-mediated CAV1 on the recruitment and polarization of neutrophils in PMN. We explored the mechanisms by which sEVs-mediated CAV1 promotes lung metastasis and neutrophil phenotypic transformation in pulmonary PMN by *in vivo* and *in vitro* experiments, intending to provide new therapeutic ideas for BC lung metastasis.

## Materials and Methods

### Chemicals and reagents

Polyclonal antibodies against CAV1, ITGB4, FAK, Phospho-FAK, Src, Phospho-Src, E-cadherin, N-cadherin, vimentin, TLR4, NF-κB p65, p-NF-κB p65, GAPDH and NLRP3 were purchased from Affinity Biosciences (Cincinnati, USA). Monoclonal antibody against ITGA6 were purchased from Abcam (Cambridge, UK). Anti-PI3K and anti-Phospho-FAK were purchased from Abmart (Shanghai China). Anti-NOS2, Anti-CD206 and anti-CD34 were purchased from Proteintech (Wuhan China). NF-κB inhibitor BAYN11-7082 and NLRP3 inhibitor MCC 950 were purchased from Selleck (Shanghai, China). Hoechst stain and Wright-Giemsa Stain were purchased from Boster Biological Technology (Beijing, China). Caveolin1 Y14 phosphorylation was inhibited by the SRC family kinase inhibitor PP2, which was obtained from Abmole Bioscience Inc (Houston, USA). Cell Counting kit (CCK-8) were purchased from Yaesen Biotechnology (Yaesen, Shanghai, China). Small interfering RNA and overexpressed pc DNA for transient transfection, as well as recombinant lentiviruses knocking down and overexpressing caveolin1 for the construction of lentiviral stably transfected cell lines, were purchased from Gemma Genetics (Suzhou, China). The liposome transfection reagent lipo3000 was purchased from Thermo Fisher Scientific (Massachusetts, USA). Agarose gel beads were purchased from MedChemExpress LLC (MCE, New Jersey, USA).

### Cell lines and cell culture

MDA-MB-231 human BC cell lines, dHL-60 human acute promyelocytic leukemia cell lines and BEAS-2B human normal lung epithelial cells were obtained from the Shanghai Cell Bank, Chinese Academy of Sciences (Shanghai, China). The cells were cultivated at a constant temperature of 37 °C in a 5% CO_2_ environment in Dulbecco's modified Eagle medium (DMEM, Solarbio, China) or Iscove's Modified Dulbecco's medium (IMDM, GIBCO, USA) supplemented with 10% fetal bovine serum (FBS, BI, USA).

### Cell transfection

Cells were plated in 6-well plates with 2 ml complete medium the day before transfection. When the cell density reached 60%-70%, the medium was changed to serum-free medium, and at the same time, OPTIM-MEM diluted lipo3000 and OPTIM-MEM diluted siRNA, pc DNA, p3000 were added to the corresponding well. Six hours after the transfection, the media containing the transfection complex was changed to DMEM or IMDM containing 10% FBS, and the cells were allowed to incubate for another 24 to 48 hours before being employed in further investigations.

### Construction of stably transfected cell lines

Pre-experiments were conducted to assess the lentiviral multiplicity of infection (MOI) beforehand and 8×10^4^ MDA-MB-231 cells were inoculated in 24-well plates to achieve a cell density of 60-70% the next day. According to the gradient MOI values of 10, 20, 40, and 80, the amount of virus was added per well. The sh RNAs were then tested for the greatest knockdown effect using the CAV1-homo-343, CAV1-homo-661, and CAV1-781 sequences, and CAV1-homo was used for overexpression of CAV1 (Table S1), while Polybrene was also added to each well to facilitate vector entry into the cells, which was replaced with DMEM containing 10% FBS after 12-24 h of infection, and green fluorescence was observed under a fluorescence microscope. After adding puromycin to each well, cells were continually screened for a month until all freshly produced cells were MDA-MB-231 cells with stable CAV1 overexpression or knockdown.

### SEVs extraction

Cells from which sEVs were extracted were cultured in Dulbecco's modified Eagle medium (DMEM, Solarbio, China) or Iscove's Modified Dulbecco's medium (IMDM, GIBCO, USA) supplemented with 10% fetal bovine serum (FBS, BI, USA). We replaced the EV-free culture fluid when we collected the cells releasing sEVs. The supernatant of the cell culture medium was collected into a centrifuge tube and spun at 300 g for 10 minutes, 2000 g for 10 minutes, and 10,000 g for 30 minutes at 4°C to remove the cells and cellular debris. The supernatant was then put into an ultrafiltration tube and spun at 5000 g for 50 min at 4 °C. Until the liquid was condensed into a yellow viscous liquid, it was centrifuged numerous times. To identify sEVs, the liquid was solubilized in phosphate-buffered saline (PBS) after being centrifuged repeatedly until it was concentrated into a yellow viscous liquid.

### SEVs identification

SEVs morphology was evaluated using transmission electron microscopy (JEOL, Tokyo, Japan), and sEVs size was determined using nanoparticle size tracing. SEVs samples were homogeneously blown, and a small amount was dropped onto a copper grid with a diameter of 2 mm. The grid was then stained for a while with drops of tungsten phosphate, and then the image was taken with a Philips Tecnai 12 BioTwin transmission electron microscope (Pontificia Universidad Catolicade Chile, Santiago, Chile) operating at 100 kV. To determine the particle size distribution of the sEVs in each group as well as the mean particle size, sEVs samples were diluted at a ratio of 5:1000 between sEVs and PBS, and three videos lasting 30 seconds each were then captured using a nanoparticle tracking analyzer of the Nano-Sight-NS30 (Worcestershire, UK) type.

### SEVs internalization

1×10^4^ Lung-CAV1 (+) or Lung-CAV1 (-) BEAS-2B cells were seeded in a 24-well plate placed on a circular crawler sheet. SEVs labeled with PKH67 were added to the cells and incubated for 24 hours. The crawler sheet was then removed, and the cells were fixed with 4% paraformaldehyde. A quencher containing DAPI recombinant was added to block the sheet, and images were captured using laser confocal microscopy (Olympus Corporation, Tokyo, Japan).

### Proliferation assay

5×10^3^ Whether Lung-CAV1 (+) or Lung-CAV1 (-) in three separate 96-well plates, BEAS-2B cells were seeded and exposed to SEVs-free medium(blank), EVs and oeCAV1-EVs, respectively. After 24 h, 48 h, and 72 h, 10 ul of CCK-8 reagent were added to each well, and absorbance was measured using an enzyme counter at 450 nm.

### Transwell migration and matrigel invasion assays

Lung-CAV1 (+) or Lung-CAV1 (-) BEAS-2B cells were incubated with sEVs-free medium (blank), sEVs, and oeCAV1-sEVs, and then inoculated in the lower chamber with complete medium. MDA-MB-231 cells were resuspended in serum-free medium and diluted to 5 × 10^5^ cells/mL. The upper chamber was covered or not covered with matrix gel, and 200 ul of cell suspension was inoculated in the upper chamber. After 24-48 h, the cells that passed through the polycarbonate membrane were fixed with 4% paraformaldehyde, stained with 0.1% crystal violet for 30 min, and the number of migrating infiltrating cells was observed under the microscope and photographed.

### Wound-healing assay

A straight wound was drawn on a monolayer of Lung-CAV1 (+) or Lung-CAV1 (-) BEAS-2B cells using a sterile pipette tip. Cells were incubated with sEVs-free medium (blank), sEVs, and oeCAV1-sEVs for 0, 24, and 48 h, and then photographed under a microscope.

### ELISA assays

The secretion of IL-6 and CCL2 in the supernatant of dHL-60 was detected by ELISA kit for IL-6 and CCL2 (Elabscience, Wuhan, China), and an enzyme-labeled instrument saw the OD value at 450 nm.

### Flow cytometry

The polarization of dHL-60 was detected by incubating dHL-60 for 30 min with Nitric Oxide Synthase NOS2 Antibody (Proteintech, Wuhan, China) and Mannose Receptor CD206 Antibody (Proteintech, Wuhan, China) using a flow cytometer (FACS Calibur, BD Biosciences).

### Co-immunoprecipitation assay

Total cellular proteins were extracted with RIPA lysate (Applygen, China), to which PMSF (Solarbio, China) and phosphatase inhibitor (Applygen, China) were added, and the supernatant was incubated overnight at 4°C by adding the Caveolin-1 antibody (Affinity, USA) and the corresponding Ig G antibody (Proteintech, China) of the corresponding species, respectively, and then 50% of the ProteinA/G Agarose (Engibody, France) was incubated for 2-4 h to form the ProteinA/G-antigen-antibody complex, washed with PBS for 3 times, and then lysed with 2× loading buffer to prepare samples for subsequent Western blot assay.

### Western blot assay

Total cellular proteins were extracted with RIPA lysate (Applygen, China) incorporating PMSF (Solarbio, China) and phosphatase inhibitor (Applygen, China), and proteins were solubilized with 6× protein loading buffer (TransGen Biotech, China). The lysates were then subjected to 8%-12% SDS-PAGE to separate proteins of different molecular weights under the action of an electric field, and the proteins were transferred to polyvinylidene difluoride membranes using electroblotting and then closed with 5% skimmed milk powder for 2h. The membrane was incubated with the corresponding primary antibody at 4°C overnight, and then the PVDF membrane was incubated at room temperature with HRP-labeled anti-rabbit IgG of goat or HRP-labeled anti-rat Ig G of goat (Zhongshan Jinqiao Company, China) for 2 h, and chemiluminescence detection was performed using the ECL Western Blotting Detection System. Quantification was performed using ImageJ software.

### Quantitative real-time PCR analysis

Total cellular RNA was extracted using the Tranzol Up kit (TransGen Biotech, China), and RNA was used as a template for reverse transcription into cDNA. Using EasyScript® RT/RI Enzyme Mix, gDNA Remover, 2×ES Reaction Mix, and Oligo(dT) _18_ formulated into a 20ul system. RT-qPCR was performed using 2× TransStart Green qPCR SuperMix, Passive Reference Dye (50×), Forward Primer, Reverse Primer, and Nuclease-Free Water (TransGen Biotech, China) formulated into a 20ul system, and this process was carried out on a StepOne RT-qPCR machine by holding at 94°C for 30 s, then at 94°C for 5 s, at 62°C for 40 s, then at 95°C for 15 s, at 60°C for 1 min, and at 95°C for 15 s. Target genes were normalized by using GAPDH as an internal reference gene. Make sure the melting curve is single-peaked to ensure primer specificity. The relative amount of target gene mRNA was calculated using the 2-CT method, and the primer sequences for the target genes are shown in Table S2.

### Immunofluorescence staining

BEAS-2B cells in the logarithmic growth phase were seeded in 24-well plates with sterile cell crawlers, then transfected and cultured for 48 hours before being fixed with 4% polymethanol and permeabilized with 0.1% Triton X100, closed at room temperature for 2 hours, and then incubated at 4°C overnight. The nuclei were stained with DAPI, and finally the slides were fixed with fixative, observed under a fluorescence microscope, and photographed.

### Construction of mouse model

To create an *in vivo* tumor model, 200 ul of untreated normal MDA-MB-231 cells from the second pair of mammary fat pads and an equal amount of PBS were injected into six-week-old BALB/c-nu mice. The domesticated mice were then injected with various groups of BC cell sEVs for a month in a row. The experimental groups were the blank group, which did not inoculate tumor cells, the untreated normal MDA-MB-231 tumorigenic group (Model), the MDA-MB-231 tumorigenic and stable knockdown CAV1 MDA-MB-231 sEVs domestication group (shCav1-sEVs), the MDA-MB-231 tumorigenic and wild MDA-MB-231 sEVs domestication group (sEVs), and the MDA-MB-231 tumorigenic and stable overexpress CAV1 MDA-MB-231 sEVs domestication group (oeCav1-sEVs).

### H&E and Immunohistochemistry staining

After one month of domestication using each group of sEVs, paraffin sections were made from mouse lung tissue. After being dewaxed with xylene, the paraffin sections were hydrated with a gradient of alcohol concentrations, and then submerged in distilled water. The paraffin tissue sections placed in a wet box were completely covered and stained for 10 minutes using hematoxylin, rinsed in tap water, and then the sections were placed in 1% hydrochloric acid alcohol to differentiate them. Following the dropwise addition of 1% ammonia, paraffin tissue sections were coated and dyed with eosin dye for 10 min, followed by decolorization with graduated alcohol concentrations and toluene permeabilization with xylene for 10 min each. Finally, neutral resin and spotless coverslips were used to block the parts.

### Public data analysis and RNA Sequencing

The Cancer Cell Line Encyclopedia (CCLE, https://sites.broadinstitute.org//ccle/) database was used to analyze the differential expression of CAV1 and integrin α6β4 (ITGA6/ITGB4) in the normal BC cell lines MCF-10A, MCF-12A, and various BC cell lines. The interaction between CAV1 and Integrin α6β4 in BC cells was examined using the STRING (https://cn.string-db.org) protein-protein interaction networks functional enrichment analysis database. Total RNA was extracted from untreated, control MDA-MB-231 cells as well as cells that had CAV1 stably knocked down and overexpressed. RNA libraries were then created, and the RNA libraries were sequenced using the Illumina Novaseq 6000 sequencing system (http://www.illumina.com.cn/) to determine which genes were significantly differentially expressed between the three sets of data.

### Statistical analysis

Statistical analysis all data were analyzed using GraphPad Prism 8 software. Results are expressed as mean ± standard deviation of at least three independent experiments. Statistical analyses were performed using Student's test and One-Way ANOVA. p<0.05 was considered statistically significant, p<0.01 was considered statistically significant, p<0.01 was considered highly statistically significant, and NS was considered not statistically significant.

## Results

### CAV1 on BC cell-derived sEVs promotes BC lung metastasis, PMN neutrophil recruitment, N2-type polarization and lung angiogenesis

**Figure 1:**
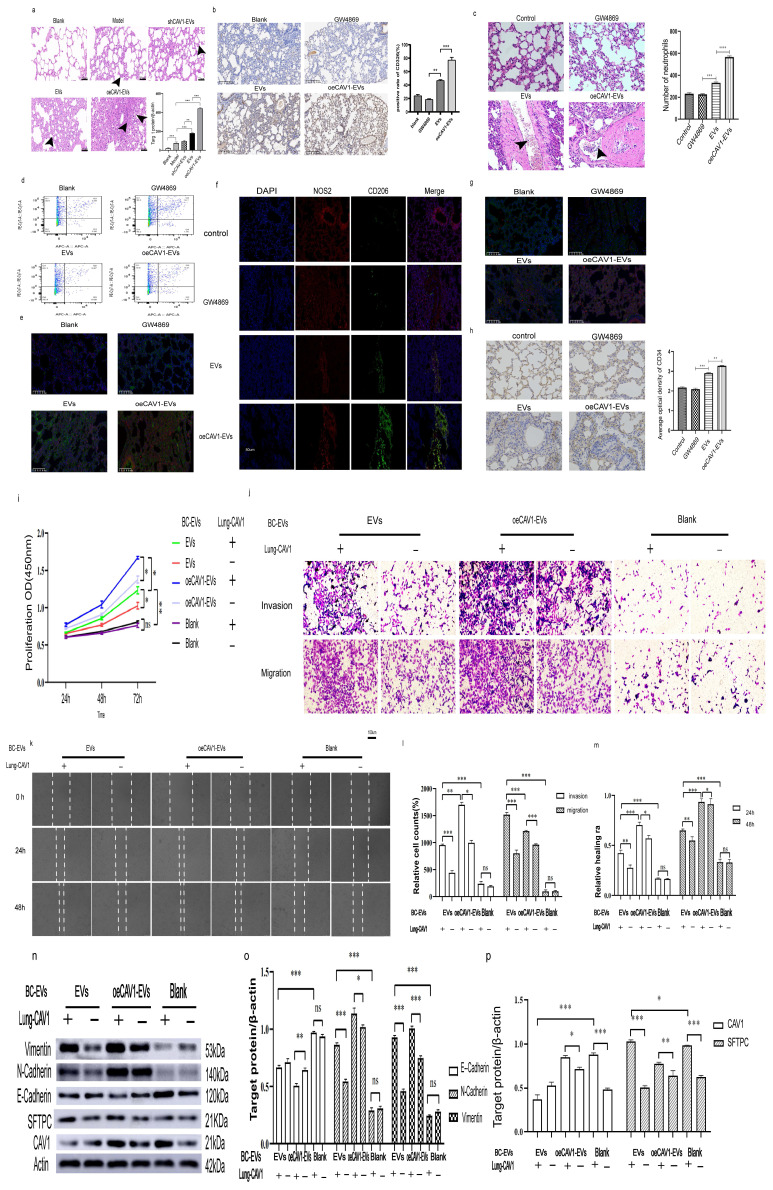
** CAV1 on BC cell-derived sEVs promotes BC lung metastasis, PMN neutrophil recruitment and N2-type polarization and lung angiogenesis.** a: HE staining to detect the effect of sEVs on lung metastasis of breast cancer in mice *in vivo*. The magnification is 400, and the arrow shows the heterogeneous proliferation of alveolar epithelial cells into papillary masses. b: IHC of lung epithelial cell marker CD326. Bar=40um. c: HE staining to detect neutrophil recruitment around breast cancer lung metastases. Bar=20um. d: Flow cytometry to test for neutrophil infiltration in lung tissue. e: Fluorescence co-localization imaging of neutrophil markers CD11b and Ly6G in the lung. f: Immunofluorescence was used to detect the expression of neutrophil polarization markers NOS2 and CD206 in each group. Bar=50um. g: Immunofluorescence of N2 neutrophil markers Ly6G and ARG2 in each group h: IHC staining was used to observe the expression of vascular endothelial marker protein CD34 in each group. Bar=100um. i: CCK8 assay was used to detect cell proliferation after co-incubation of lung epithelial cells and BC sEVs. j: Transwell was used to detect the effect of sEVs on the migratory invasion of lung epithelial cells at different levels of CAV1. Bar=100um. k: Wound healing was used to detect cell migration after co-incubation of sEVs and BC sEVs.Bar=200um. l.m: Statistical analysis of j and k. n: Western blot analysis was performed to detect the expression of EMT associated proteins after incubation of lung epithelial cells with EV and the expression of SFTPC in lung epithelial cells after incubation with EV. o, p: Statistical analysis of n. Data ware shown as mean ± SD and assessed with One-way ANOVA test. (n=3) (ns stands for non-significant difference; *p<0.05; **p<0.01; ***p<0.001).

SEVs mediate intercellular and cell-to-matrix communication by transporting their encapsulated nucleic acids and proteins. Supplementary Figure S1 shows that Cav-1 can regulate the expression of proteins on BC sEVs, and BC-derived sEVs could transport CAV1. Our previous results indicated that the alteration of CAV1 in BC cells affected the content of CAV1 in BC-derived sEVs. Next, we explored the effects of Cav-1 in BC cell-derived sEVs on BC lung metastatic tissue. We first constructed an *in vivo* tumor model by injecting BC cells into the mammary fat pads of mice and then domesticated the lung tissues by injecting BC cell-derived sEVs with different levels of CAV1 into the tail vein. Alveolar epithelial cells with large, darkly stained, anomalous nuclei and loss of polarity were visible in the HE-stained sections of lung tissues affected by sEVs. Distinctive papillary masses (indicated by arrows) and adenoma-like hyperplasia were found in the lung tissues. These are lung lesions characteristic of BC lung metastasis. Statistical analysis showed that the oeCAV1-sEVs group had the highest number of alveolar epithelial cells with the above changes and the difference was statistically significant (P<0.001), indicating that BC-derived sEVs overexpressing CAV1 can promote BC metastasis to the lungs *in vivo* (Fig. 1a). Immunohistochemistry of epithelial cell marker CD326 showed that BC-derived sEVs overexpressing CAV1 promote proliferation of lung epithelial cells and lung epithelial cells are most affected cells by BC-derived sEVs in lung tissue (Fig. 1b). We used sEVs inhibitor GW4869 to change CAV1 in BC-derived sEVs *in situ* tumor models and found that the number of recruited neutrophils was much higher in oeCAV1 sEVs group. We could observe BC lung metastatic cells around the recruited neutrophils in these lung tissue sections. Besides, the effect of CAV1 on neutrophil recruitment was also confirmed by a significant reduction in lung infiltration of neutrophils in the sEVs inhibitor group (GW4869 group) (Fig. 1c). Through flow cytometry analysis, we found the increased infiltration of neutrophils in lung tissue in the pc-CAV1 group (Fig. 1d). Fluorescence co-localization imaging of neutrophil markers CD11b and Ly6G indicate increased neutrophil infiltration by BC-derived sEVs overexpressing CAV1 (Fig. 1e). It also showed increased expression of the N1-type marker inducible nitric oxide synthase NOS2 in the lungs of the sEVs inhibitor group (GW4869 group) compared with the EV group, whereas the expression of the N2-type marker mannose receptor CD206, Ly6G andARG2 was increased in the sEVs group of oeCAV1 (Fig. 1f-g). Immunohistochemistry of the pulmonary vascular endothelial marker protein CD34 showed decreased expression of CD34 in the GW4869 group and increased expression of CD34 in the sEVs group of oeCAV1 compared with the sEVs group (Fig. 1h). All the above data indicate that CAV1 on BC sEVs overexpressing CAV1 promotes BC metastasis to the lungs *in vivo*, promotes neutrophil recruitment and N2-type polarization around lung metastatic site, and promotes angiogenesis in the lungs.

In addition to experiments *in vivo*, we also explored the effects of CAV1 on lung epithelial cells at the *in vitro* cellular level. We hypothesized that CAV1 arriving in the lungs could alter the characteristics of lung epithelial cells, thereby creating an environment conducive to BC lung metastasis. We incubated lung epithelial cells containing CAV1 and those knocked down of CAV1 with sEVs from MDA-MB-231 cells stably knocking down and overexpressing CAV1, respectively, and observed changes in lung epithelial cell BEAS-2B proliferation, invasion, migration, and EMT. As shown in supplementary Figure S2, CAV1-homo-788 was most effective in reducing CAV1 expression. The results of the proliferation assay, Transwell invasion experiments, and Wound-healing tests indicated that the proliferation, migration ability, and invasion ability of BEAS-2B cells through the holes on the matrix gel and chambers were enhanced with the increase of CAV1 in sEVs. In addition, down-regulation of CAV1 in lung epithelial cells could attenuate the promotional effect of CAV1 in BC sEVs on BEAS-2B cell proliferation, migration and invasion (Fig. 1i-m). Western blot showed that in the presence of CAV1 in lung epithelial cells, EVs and oeCAV1-sEVs group in comparison with blank group showed decreased expression of E-Cadherin and increased expression of N-Cadherin and Vimentin, suggesting the promotion of EMT; knockdown of CAV1 in lung epithelial cells could attenuate the promotion of EMT in BEAS-2B cells by CAV1 in sEVs (Fig. 1n-p). In our animal experiments, distinct papillary nodules could be seen in HE-stained sections of the lungs, which are lung lesions with features of lung metastasis from BC (Fig 1a). The above experiments suggest that CAV1 creates a microenvironment that facilitates BC lung metastasis by transforming lung epithelial cells into a pathological state. In conclusion, CAV1 on BC cell-derived sEVs could promote BC metastasis to the lungs, enhance the recruitment and polarization of neutrophils around lung metastatic foci, increase angiogenesis in the lungs *in vivo*, and creating a microenvironment favorable for BC lung metastasis by altering the state of lung epithelial cells. Knockdown of CAV1 in BC cells or lung epithelial cells could attenuate the promotional effects of BC sEVs on the proliferation, invasive migration, and EMT of lung epithelial cells.

### CAV1 promotes BC cell sEVs internalization in lung epithelial cells by regulating SFTPC expression on lung epithelial cells

**Figure 2:**
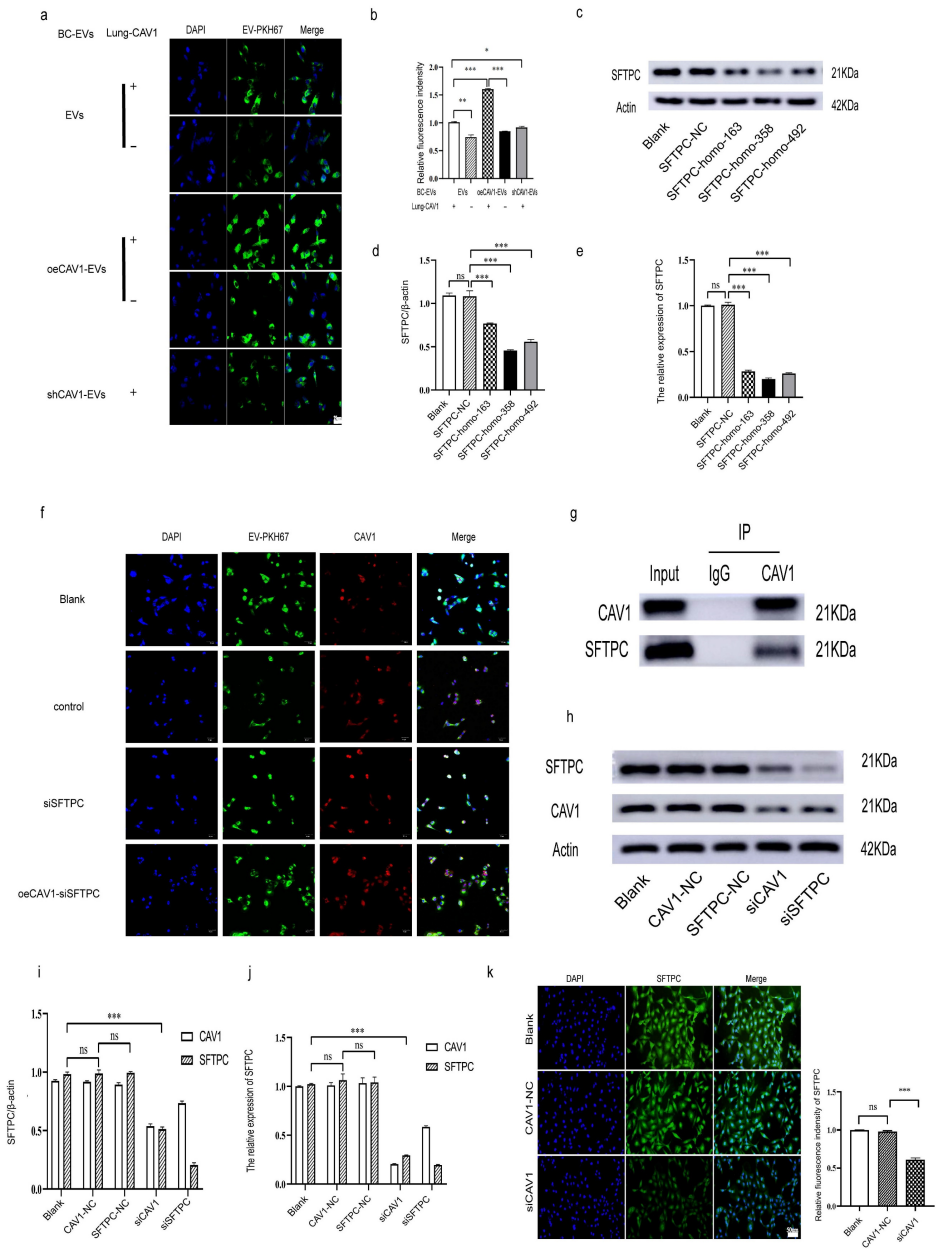
** CAV1 promotes BC cell sEVs internalization in lung epithelial cells by regulating SFTPC expression on lung epithelial cells.** a: Laser confocal microscopy was used to detect the internalization of PKH67-labeled sEVs in lung epithelial cells. Bar=25um. b: Statistical analysis of a. c-e: Western blot and RT-qPCR were performed to detect the siRNA fragment that knocked down the SFTPC most significantly in BASE-2B. f: Immunofluorescence experiment to detect the BC-derived sEVs internalization in BEAS-2B cell. g: Immunoprecipitation was used to detect the interaction between CAV1 and SFTPC in BASE-2B. h-j: Western blot and RT-qPCR were performed to detect the expression of SFTPC after knockdown of CAV1 in BASE-2B, and the expression of CAV1 after knockdown of SFTPC, k: Immunofluorescence was used to detect the expression of SFTPC after knockdown of CAV1 in BASE-2B. Data ware shown as mean ± SD and assessed with One-way ANOVA test. (n=3) (ns stands for non-significant difference; *p<0.05; **p<0.01; ***p<0.001).

To investigate how CAV1 causes the above changes in BEAS-2B cells, we next explored the effects of Cav-1 on BC-derived sEVs and CAV1 on lung epithelial BEAS-2B cells on the uptake of BC sEVs by BEAS-2B cells. We extracted sEVs from MDA-MB-231 cells with stable knockdown or overexpression of CAV1 and sEVs from MDA-MB-231 cells with regular CAV1 expression and incubated BEAS-2B cells which is with or without CAV1 after staining BC sEVs using PKH67. Confocal microscopy showed that with unchanged CAV1 in the lung epithelium, the BEAS-2B cells showed the highest uptake of BC sEVs overexpressing CAV1. Reducing CAV1 on BC cell-derived sEVs and decreasing CAV1 in lung epithelial cells resulted in fewer sEVs uptake by lung epithelial cells, suggesting that altering CAV1 on BC cell-derived sEVs and CAV1 expression in lung epithelial cells affects sEVs uptake by BEAS-2B cells (Fig. 2a-b). SFTPC is a recognition protein for integrin α6β4 on BC sEVs in the lung. We transfected three siRNA sequences in BEAS-2B cells to screen out the best sequences for knocking down SFTPC. As shown in Fig. 2c-e, SFTPC-homo-358 was the most effective in reducing the expression of SFTPC at both protein and mRNA knockdown levels, so we chose SFTPC-homo-358 for the subsequent experiments. Then we knocked down SFTPC in BEAS-2B cells and found lung epithelial cells' decreased uptake of breast cancer-derived sEVs. Overexpression of CAV1 in MDA-MB-231 cells based on knockdown of SFTPC increases sEVs uptake compared with the KO SFTPC group (Fig. 2f). Fig. 1n showed that the knockdown of CAV1 in BEAS-2B cells decreased SFTPC expression in lung epithelial cells, which inhibited the uptake of BC sEVs by BEAS-2B cells. To further illustrate the relationship between CAV1 and SFTPC in BEAS-2B cells, we performed immunoprecipitation experiments in lung epithelial cells to detect whether CAV1 and SFTPC interacted. Additionally, we knocked down CAV1 and SFTPC in BEAS-2B cells, and Western blot and RT-qPCR were performed to detect the regulatory relationship between them.

In addition, we used immunofluorescence to see the fluorescence intensity of SFTPC after the knockdown of CAV1 in BEAS-2B cells. We used an antibody to CAV1 to de-precipitate the protein complexes and then performed a Western blot to verify that the CO-IP results showed that CAV1 could co-precipitate with SFTPC (Fig. 2g). Both Western blot and RT-qPCR results showed that knockdown of CAV1 could significantly down-regulate the expression of SFTPC; knockdown of SFTPC could also down-regulate the expression of CAV1, only the difference was not as significant as the decrease of SFTPC by CAV1 (Fig. 2h-j). Immunofluorescence results showed that the fluorescence intensity of cells in the knockdown CAV1 (siCAV1) group was significantly decreased compared with the Blank group and the null-loaded (CAV1-NC) group (Fig. 2k). All of the above results indicated that CAV1 interacts with SFTPC, and knockdown of CAV1 could down-regulate the expression of SFTPC. Taken together, we concluded that CAV1 in BEAS-2B cells promotes the internalization in BC cell sEVs by lung epithelial cells by regulating the expression of SFTPC.

### CAV1 promotes the expression of integrin α6β4 to facilitate the internalization in BC cell sEVs by lung epithelial cells

**Figure 3:**
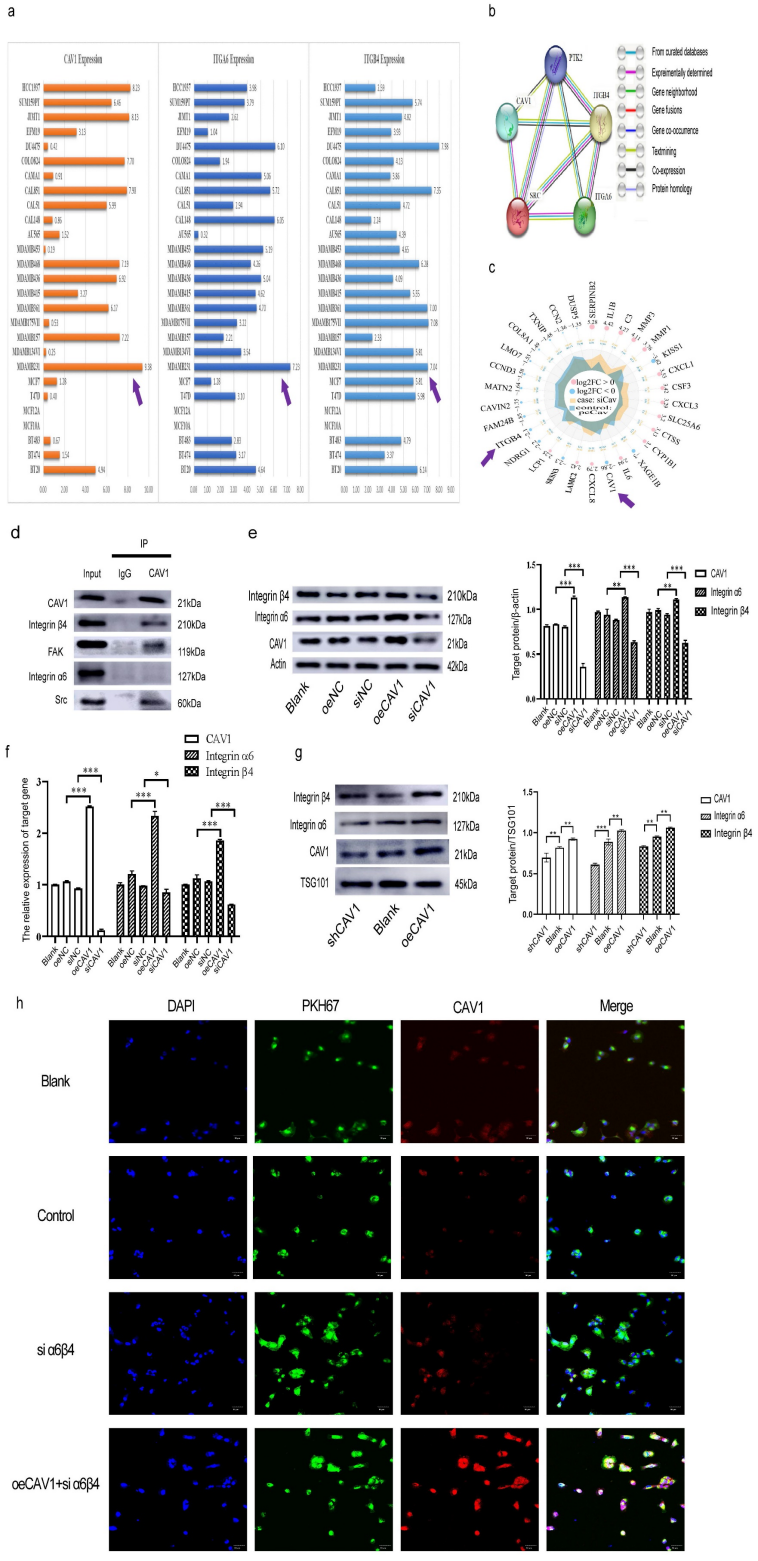
** CAV1 promotes the expression of integrin α6β4 to facilitate the internalization in BC cell sEVs by lung epithelial cells.** a: The CCLE database was used to analyze the expression of CAV1 and integrin α6β4 in different BC cell lines. b: STRING database was used to analyze the PPI network maps of CAV1, ITGA4, ITGB4, FAK, and Src. c: Radar plots of differential genes following knockdown and overexpression of CAV1 in MDA-MB-231. d: Immunoprecipitation was used to detect interactions of CAV1 with ITGA4, ITGB4, FAK, and Src. e: Western blot analysis was performed to detect the expression of integrin α6β4 after interference with CAV1 in MDA-MB-231 cells. f: RT-qPCR was performed to detect the expression of MDA- MB-231 cells after interfering with CAV1 expression of integrin α6β4. g: Western blot analysis was performed to detect CAV1 regulation of integrin α6β4 expression on BC EV. h: Immunofluorescence was utilized to detect uptake of BC-derived sEVs by BESE-2B cells. Data ware shown as mean ± SD and assessed with One-way ANOVA test. (n=3) (ns stands for non-significant difference; *p<0.05; **p<0.01; ***p<0.001).

Our previous results suggest that both CAV1 on BC cell sEVs and CAV1 on lung epithelial cells affect the internalization in BC sEVs by BEAS-2B cells, thereby promoting the oncogenic progression of BEAS-2B. CAV1 in BEAS-2B cells encourages the internalization of BC cell sEVs in the lung epithelium by regulating the expression of SFTPC. Since SFTPC is a recognition protein for integrin α6β4 on BC sEVs in the lung, and integrin α6β4 on sEVs binds to SFTPC in lung epithelial cells, resulting in sEVs translocation to the lung^26^, we hypothesized that CAV1 may promote the internalization in BC sEVs by BEAS-2B cells by regulating the expression of integrin α6β4 on BC cells and sEVs. To verify this idea, we first analyzed the expression of CAVI, ITGA6, and ITGB4 in different BC cell lines in The Cancer Cell Line Encyclopedia database. We used online tools to explore the relationship between CAV1, ITGA6, and ITGB4 on the STRING database. In addition, we performed RNA sequencing by Illumina Novaseq 6000 sequencing system using stably transfected cell lines with stable knockdown and overexpression of CAV1 to screen for differential genes with altered CAV1 expression levels. CCLE database analysis showed that CAV1 and integrin α6β4 were highly expressed in triple-negative breast cancer (TNBC) MDA-MB-231 cells (Fig. 3a). PPI network diagrams of protein interactions showed that CAV1 interacted with ITGB4, Src, and FAK (PTK2), with CAV1 and ITGB4 genes neighboring as well as co-expressed, suggesting functional relevance (Fig. 3b). Radar plots of RNA sequencing showed that ITGB4 was expressed consistently with CAV1. CAV1 down-regulated (log2FC=-2.86), and ITGB4 expression was also significantly down-regulated (log2FC=-2.1) (Fig. 3c). CO-IP results showed that CAV1 co-precipitated with Integrin β4 but negative with integrin α6 in MDA-MB-231 cells, and FAK and Src were also precipitated by CAV1 (Fig. 3d). It suggests that CAV1 has interactions with Integrin α6β4, especially with its functional subunit Integrin β4, while the relationship with the regulatory subunit Integrin α6 may be manifested through Src and FAK kinase. To further verify the relationship between CAV1 and Integrin α6β4, we extracted proteins and RNA from MDA-MB-231 cells for Western blot and RT-qPCR to detect the expression of integrin α6β4 after interfering with CAV1 in MDA-MB-231 cells and also extracted sEVs of MDA-MB-231 cells, and Western blot was performed to detect the expression of integrin α6β4. Both Western blot and RT-qPCR results showed that the expression of integrin α6β4 was increased after overexpression of CAV1, and the expression of integrin α6β4 was decreased after knockdown of CAV1 (Fig. 3e-f). Western blot results of sEVs showed the same trend (Fig. 3g). To confirm the role of integrins for sEVs uptake, we knocked down the expression of integrins on BEAS-2B cells and found a reduction in sEVs uptake in recipient cells. The more CAV1 was present in sEVs, the more CAV1 was taken up by BEAS-2B cells (Fig. 3h). In summary, we conclude that CAV1 in BC cells promotes integrin α6β4 expression, supporting the internalization of BC cell sEVs by lung epithelial cells.

### CAV1 Promotes BC Lung Metastasis by Activating the Src/FAK/α6β4 Pathway in MDA-MB-231 Cells and Simultaneously Activating Src/PI3K Signaling Downstream of α6β4 in Lung Epithelial Cells

**Figure 4:**
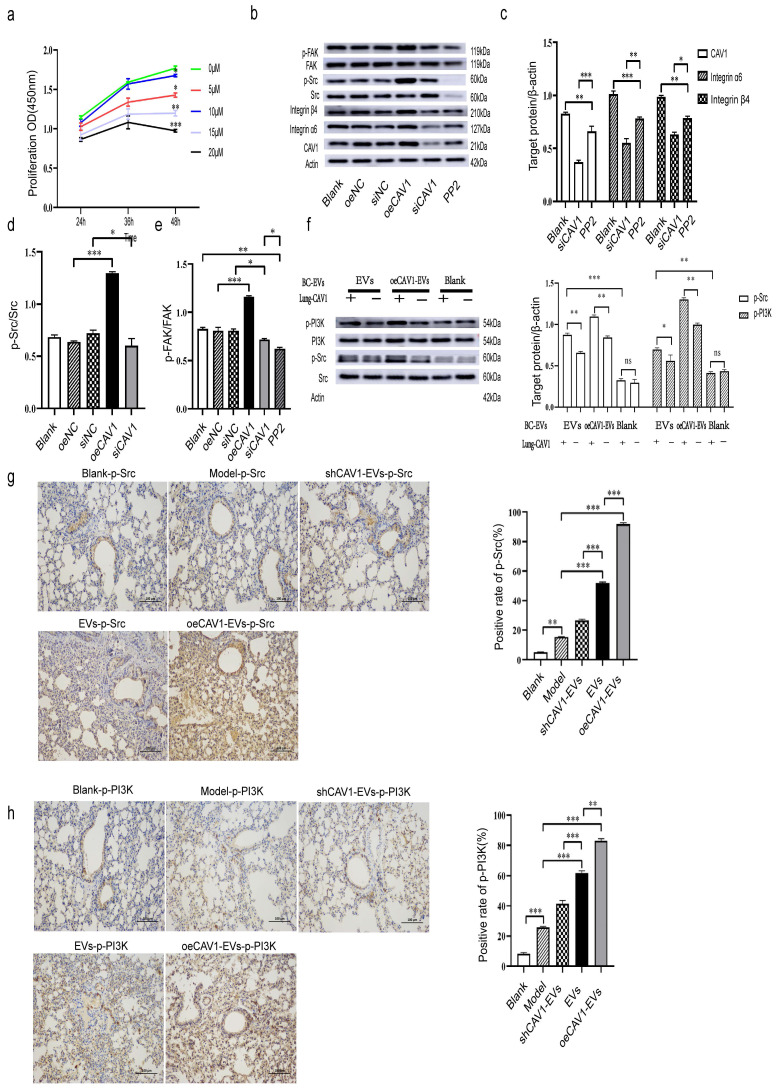
** CAV1 Promotes BC Lung Metastasis by Activating the Src/FAK/α6β4 Pathway in MDA-MB-231 Cells and Simultaneously Activating Src/PI3K Signaling Downstream of α6β4 in Lung Epithelial Cells.** a: CCK8 assay was used to detect the optimal acting concentration of the Src inhibitor PP2. b-e: Western blot analysis was performed to detect the expression of CAV1 and integrin Src/FAK/α6β4 signaling pathway after overexpression, knockdown, and addition of PP2. f: Western blot was used to detect the activation of integrin α6β4 downstream signaling in lung epithelial cells. g,h: IHC staining was used to detect the activation of integrin α6β4 downstream signaling PI3K/Src in mice lungs. Bar=100um. Data ware shown as mean ± SD and assessed with One-way ANOVA test. (n=3) (ns stands for non-significant difference; *p<0.05; **p<0.01; ***p<0.001).

We next explored whether CAV1 regulates the expression of integrin α6β4 through activation of Src/FAK. We first detected the expression of p-Src and p-FAK by Western blot after interfering with CAV1 expression in MDA-MB-231 cells. Then, we used the Src inhibitor PP2, and we screened the optimal concentration of PP2 at 10 uM using CCK8 assay to minimize the effect of the inhibitor on cell proliferation (Fig. 4a). Western blot results showed that overexpression of CAV1 increased the values of p-Src/Src and p-FAK/FAK, and knockdown of CAV1 decreased the values of p-Src/Src and p-FAK/FAK, and the addition of PP2 also had an effect on FAK phosphorylation (Fig. 4b-e). All this suggested that CAV1 regulates integrin α6β4 expression by activating Src/FAK. Furthermore, we found that inhibition of CAV1 suppressed EMT in MDA-MB-231 cells (Fig. S3). Some publications have reported that p-Src and p-PI3K are essential for lung metastasis in breast cancer 35-37. Nie *et al.* reported that BC cell-derived integrin α6β4 recognizes lung epithelial SFTPC and further activates the downstream PI3K/AKT signaling pathway and phosphorylation of Src to promote malignant cell proliferation and invasion 30. Next, we incubated lung epithelial cells (with or without CAV1) with BC-derived sEVs of different CAV1 expression levels and extracted proteins from each group of cells. Western blot results showed that the more CAV1 in BC sEVs, the higher the levels of p-Src and p-PI3K, under the condition of the unchanged expression level of CAV1 in lung epithelium; incubation of BEAS-2B cells with BC sEVs that overexpress CAV1, p-Src and p-PI3K were reduced in the knockdown CAV1 group in lung epithelial cells compared with BEAS-2B cells typically expressing CAV1 (Fig. 4f). In addition, we validated this pathway in an animal model. We took lung tissues from mice domesticated with sEVs with different CAV1 contents after planting tumor cells, and immunohistochemical staining was performed to detect the activation of Src/PI3K downstream of integrin α6β4 in the lungs of mice. The results showed elevated expression of p-Src and p-PI3K in lung tissues of the oeCAV1-sEVs group compared to the sEVs group and decreased expression of p-Src and p-PI3K in lung tissues of the shCAV1-sEVs group (Fig. 4g-h). Taken together, we suggest that CAV1 promotes BC lung metastasis by activating the Src/FAK/α6β4 pathway in MDA-MB-231 cells and simultaneously activating Src/PI3K signaling downstream of α6β4 in lung epithelial cells.

### CAV1 in BC-derived sEVs promotes neutrophil recruitment, secretes IL-6 and CCL2, promotes N2-type polarization, and increases VEGF-A and MMP9 expression

**Figure 5:**
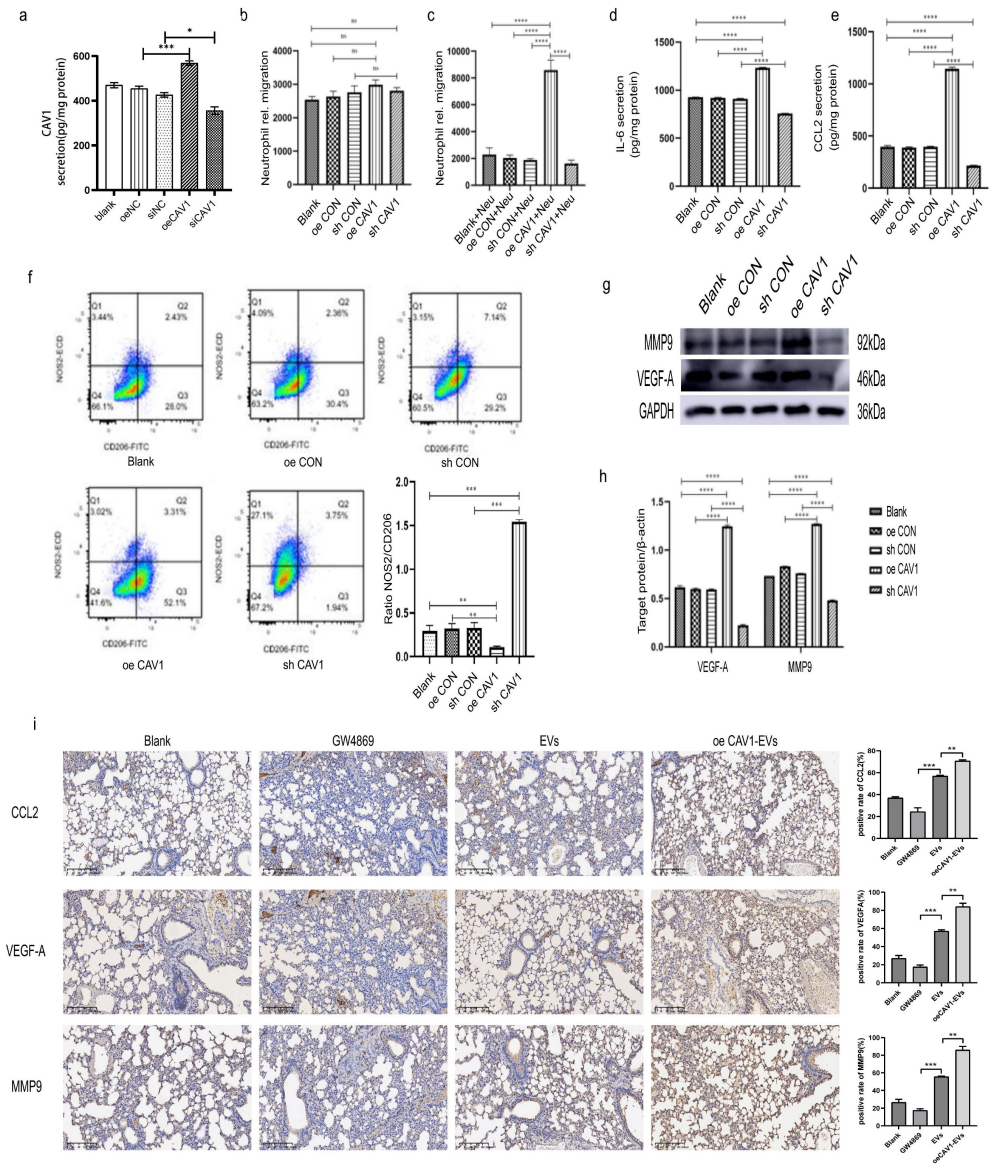
** CAV1 in BC-derived sEVs promotes neutrophil recruitment, secretes IL-6 and CCL2, promotes N2-type polarization, and increases VEGF-A and MMP9 expression *in vitro*.** a: ELISA results of MDA-MB-231 cell supernatants containing different levels of CAV1 alone in each group. b: Dual chamber migration assay illustrated that there was no significant difference in the effect of CM on the migratory ability of dHL-60 in each group of cells. c: Dual chamber migration assay illustrated that pretreatment with oeCAV1-containing CM significantly increased the migratory ability of dHL-60 in each group. d,e: ELISA assay was used to detect IL-6 and CCL2 secretion. f: Flow cytometry was used to analyze the polarization of dHL-60. g: Western blot analysis was performed to detect the expression of dHL-60 VEGF-A and MMP9. h: Statistical analysis of g. i: IHC images demonstrated the expression of CCL2, VEGF-A and MMP9 in lung tissue. Data ware shown as mean ± SD and assessed with One-way ANOVA test. (n=3) (ns stands for non-significant difference; *p<0.05; **p<0.01; ***p<0.001).

We carried on exploring the mechanisms of CAV1 in BC-derived sEVs on neutrophil recruitment, N2-type polarization, and angiogenesis *in vitro*. HL-60 was induced into neutrophil-like dHL-60 using ATRA, and HL-60-induced differentiation was examined by Hoechst and Wright-Giemsa staining. According to Hoechst staining results, 2uM of ATRA for 5 days and 3uM of ATRA for 4 days had the best results, and the cells became multiple nuclei, which were similar to the characteristics of neutrophils. Wright-Giemsa Stain showed that the cells induced by 3uM of ATRA for 4 days had multiple nuclei compared with the normal HL-60 cells, whereas the control HL-60 cells had single nuclei. RT-qPCR was performed to detect the expression of neutrophil surface marker CD11b and we found that the mRNA level of CD11b was significantly higher in the induced group compared with the control group (Fig. S4A-C). All of the results above suggest that induction with 3uM ATRA for 4 days can induce HL-60 to dHL-60. We did an ELISA assay to detect CAV1 in the supernatant of each group of MDA-MB-231 cells (Fig. 5a). We next stimulated dHL-60 with supernatants from various groups of MDA-MB-231 cells containing different levels of CAV1, and the results of migration experiments showed that more dHL-60 cells were recruited in the oeCAV1 group (Fig. 5b-c). ELISA was conducted to detect the levels of chemokines IL-6 and CCL2 in the supernatant of dHL-60. As demonstrated in Fig. 5d-e, the secretion of both IL-6 and CCL2 increased after overexpression of CAV1, whereas a decrease in CAV1 levels showed the opposite trend. We next incubated dHL-60 with BC-derived sEVs with distinct expression levels of CAV1. Flow cytometry results showed that the ratio of NOS2^+^/CD206^+^ was reduced when dHL-60 was incubated with supernatants from MDA-MB-231 cells that were overexpressing CAV1. In contrast, the ratio was enhanced when dHL-60 was incubated with supernatants from MDA-MB-231 cells that were knocking down CAV1 (Fig. 5f). We examined the expression of VEGF-A and MMP9 with the same treatments to assess the level of angiogenesis. We found that the expression levels of VEGF-A and MMP9 rose with the increase of CAV1 expression and dropped with the decrease of CAV1 expression (Fig. 5g-h). In *in vivo* experiments, we demonstrated that neutrophils can take up sEVs of BC origin, and the expression of VEGFA, MMP9 and CCL2 *in vivo* follows the same trend as *in vitro* (Fig. 5i). In conclusion, CAV1 in BC sEVs could promote the migration and N2-type polarization of dHL-60, the expression of VEGF-A and MMP9, and the secretion of IL-6 and CCL2 by dHL-60, thus promoting BC lung metastasis.

### CAV1 in BC-derived sEVs promote neutrophil recruitment through the TLR4-NF-κB-IL-6/CCL2 axis and neutrophil N2 polarization through the TLR4/NF-κB/NLRP3 pathway

**Figure 6:**
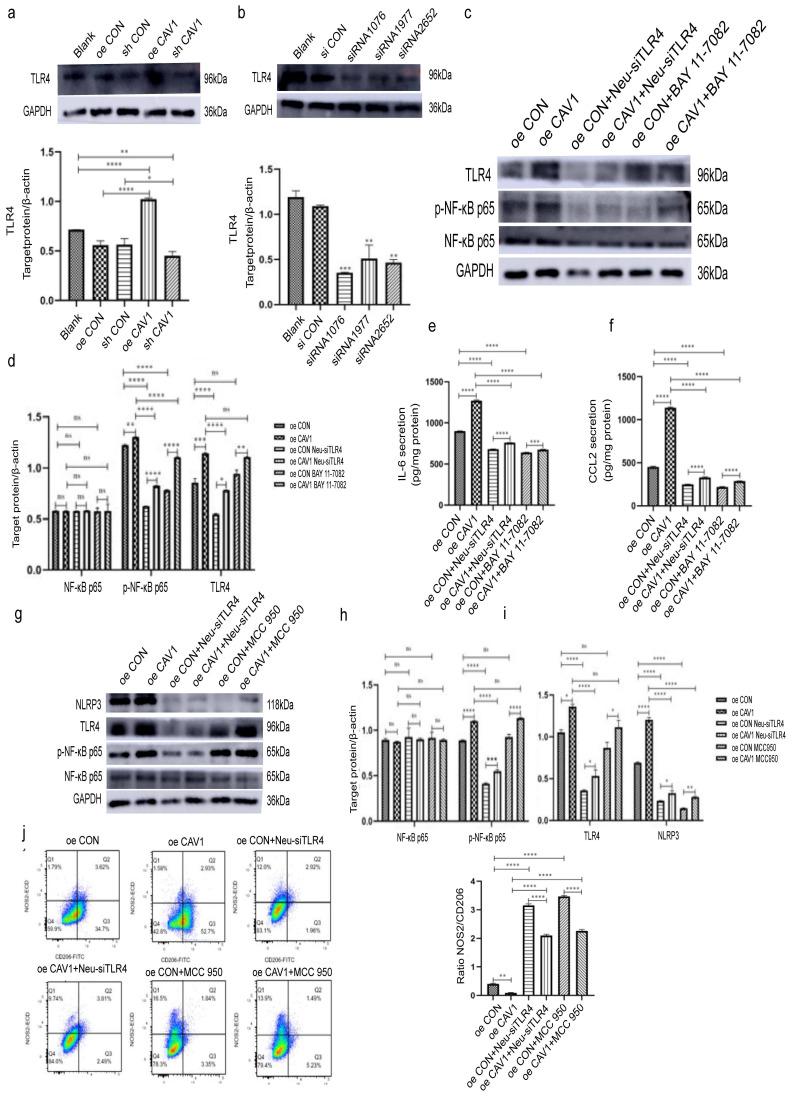
** CAV1 in BC-derived sEVs promote neutrophil recruitment through the TLR4-NF-κB-IL-6/CCL2 axis and neutrophil N2 polarization through the TLR4/NF-κB/NLRP3 pathway.** a: Western blot was performed to detect the effect of sEVs containing different CAV1 on the expression level of dHL-60 TLR4. b: Western blot was performed to detect the siRNA fragment that knocked down the TLR4 most significantly in dHL-60. c: Western blot was used to detect the expression levels of p-NF-κB p65 and TLR4 in each group of dHL-60. d: Statistical analysis of c. e,f: ELISA was used to detect the secretion of IL-6 and CCL2 in each group of dHL-60. g: Western blot analysis was performed to detect the expression of dHL-60 p-NF-κB p65, TLR4, and NLRP3 in each group. h,i: Statistical analysis of g. j: Flow cytometry analysis of dHL-60 polarization in each group. Data ware shown as mean ± SD and assessed with One-way ANOVA test. (n=3) (ns stands for non-significant difference; *p<0.05; **p<0.01; ***p<0.001).

To explore the specific mechanism by which BC sEVs caused the above changes in dHL-60, we first examined TLR4 expression in dHL-60 after incubation with sEVs with different CAV1 levels. We observed that TLR4 expression was elevated after treatment with sEVs overexpressing CAV1, while a decrease in the level of CAV1 showed the opposite tendency (Fig. 6a). Next, we selected siRNA1076 as the most effective in reducing TLR4 expression at the protein level and knocked down TLR4 in dHL-60 (Fig. 6b). Subsequently, we incubated dHL-60 with sEVs containing different levels of CAV1 and added NF-κB inhibitor BAY 11-7082. Western blot and ELISA revealed that after treatment with overexpressing CAV1 sEVs, the expression of p-NF-κB p65 and the secretion of IL-6 and CCL2 were increased in dHL-60 while the level of total NF-κB p65 expression was unchanged. Knockdown of TLR4 in dHL-60 after incubating oeCAV1 sEVs inhibited the promotion of p-NF-κB p65 expression and increased IL-6 and CCL2 secretion by CAV1. Adding BAY 11-7082 in the same incubation has similar effects (Fig. 6c-f). It suggests that sEVs containing CAV1 facilitate lung neutrophil recruitment through TLR4- NF-κB-IL-6/CCL2 axis. Similarly, we found that the expression of p-NF-κB p65 and NLRP3 in dHL-60 elevated after the incubation of oeCAV1 sEVs and unchanged NF-κB p65 expression. Knockdown of TLR4 upon addition of oeCAV1 sEVs similarly inhibited CAV1 promotion of increased p-NF-κB p65 and NLRP3 expression and NLRP3 inhibitor MCC 950 showed the same outcomes (Fig. 6h-i). Besides, flow cytometry results demonstrated that before incubating dHL-60 with sEVs overexpressing CAV1, the use of either the siTLR4 plasmid or the NLRP3 inhibitor MCC 950 inhibited the CAV1-promoting effect on the reduction of NOS2^+^/CD206^+^ values (Fig. 6j). So, we conclude that CAV1 in BC-derived sEVs promote neutrophil recruitment through the TLR4-NF-κB-IL-6/CCL2 axis and neutrophil N2-type polarization through the TLR4/NF-κB/NLRP3 pathway, thus promoting the lung metastasis of breast cancer.

## Discussion

TNBC is considered the most aggressive subtype of BC characterized by short survival, partly due to the lack of effective targeted therapies. The vast majority of mortality is due to metastasis to distant organs such as lungs and bones 38. Secreted regulators expressed in the tumor microenvironment modulate or act as mediators that together determine the direction of tumor metastasis 39. Identification of regulatory factors in the determinants of BC lung metastasis will provide potential prognostic markers and therapeutic targets.

Although CAV1 expression was significantly higher in normal breast tissues than in cancerous tissues according to the TCGA database, the conclusion that CAV1 inhibits cancer in the early stage and promotes cancer in the late stage remains valid 40. SEVs secreted by cancer cells recognize and bind to the cells at the target metastatic site specifically thus activating tumor metastatic signals and promoting the distant metastasis 41. It is demonstrated that intracellular CAV1 regulates the formation of sEVs and secretion selection 12, and upregulating CAV1 expression increases sEVs uptake 42. Previous study of our team illustrated that CAV1 can be transported to lung through BC-derived sEVs^18^. In this study, we found distinctive papillary masses and adenoma-like hyperplasia in the lung tissues of in-situ BC model after injecting CAV1-containing sEVs. We changed the expression of CAV1 and found overexpressing CAV1 can promote BC metastasis to the lungs *in vivo* overexpressing CAV1 can promote BC metastasis to the lungs *in vivo*. Those indicate that CAV1 encapsulated by sEVs promote lung metastasis.

Integrins are important cell surface receptors that mainly mediate cell adhesion to the extracellular matrix. A study published in Nature indicated that integrin α6β4 was highly expressed in sEVs promoting lung metastasis 26. Different integrins are encapsulated in sEVs of distinct tumor origins and play a role in targeting transportation to specific organs 11. A current study found that the expression of relevant integrins in lung cancer cells is suppressed along with the reduction of CAV1^43^. In this study, we analyzed that integrin α6β4 was highly expressed in the TNBC cell line MDA-MB-231 using bioinformatics and that integrin α6β4, as a pro-lung molecule, promotes lung metastasis of TNBC. Our study reveals for the first time the regulatory relationship between CAV1 and integrin α6β4 in BC predisposition to lung metastasis. We also found that the expression of integrin signaling molecules Src and FAK was also positively correlated with CAV1. CAV1 is upstream of integrin signaling and directly regulate integrin β4 or regulate the expression of integrin α6β4 through Src and FAK. The CAV1/Src/ FAK/ integrin α6β4 signaling regulatory mechanism will also provide a reference for Src inhibitor treatment of triple-negative breast cancer 44.

Lung epithelial cells are the main cell type in lung tissue. BC-derived CAV1 is transported by sEVs to the lungs, where it recognizes and binds to lung epithelial cells, transforming them into pathological states. Our study demonstrated that CAV1-containing sEVs enhanced the proliferation, invasion, and migration capacity of lung epithelial cells and promoted EMT. Alterations in lung epithelial cells provide a suitable environment for lung metastasis of breast cancer. SFTPC is an important recognition protein on lung epithelial cells. In our study, we knocked down SFTPC on the surface of BEAS-2B cells and found that the uptake of sEVs was reduced, showing the importance of SFTPC for the recognition and uptake of sEVs by lung epithelial cells.

In these years, TANs have attracted more attention in tumor metastasis. Researches have demonstrated that neutrophil infiltration, polarization and neutrophil extracellular trap formation are beneficial for BC lung metastasis 38, 45-47. It has been shown that TANs in mice are marked by CD11b(+)/Ly6G(+) 48. We then used flow cytometry and immunofluorescence co-localization imaging to show that BC-derived sEVs increase CD11b(+)/Ly6G(+) TAN infiltration in lung tissue. Studies have found that N1 TAN can be identified by the marker NOS2, while N2 TAN can be identified with the marker CD206^49^. We used flow cytometry and immunofluorescence experiments to find that pc CAV1 sEVs stimulated dHL-60 had higher expression of CD206, while shCav-1 sEVs stimulated dHL-60 had higher expression of NOS2, suggesting that oeCAV1 sEVs promote the N2-type polarization of dHL-60.

CAV1 interact with Toll-like receptors (toll-like receptors, TLRs) and immune cells. Mizuno *et al.* found CAV1 can interact with TLRs and regulate the inflammatory process associated with sepsis by binding to TLR-9 on neutrophils 50. It has been shown that the release of inflammatory factors such as interleukin 1β, interleukin 6, and tumor necrosis factor α can be significantly reduced by inhibiting the TLR-4/MyD88/NF-κB signaling pathway in a CAV1-deficient mouse model 33, 51-53. Antuamwine *et al.* suggested that increased expression of VEGF, MMP9 and CCL2 is a reflection of the N2 phenotype 54. Therefore, we speculated whether CAV1 could influence the release of chemokines IL-6 and CCL2 through the TLR4/NF-κB pathway. Our *in vivo* experiments also confirmed increased expression of CCL2, VEGF-A and MMP9, flanking the N2 polarization of the TANs. NLRP3 inflammasome is a complex of multiple proteins that are central components of the immune and inflammatory response 55. A recent study demonstrated that tumor-derived exosomal TRIM59 activates NLRP3 inflammasomes in macrophages and promotes their pro-tumor M2 polarization, thereby promoting lung cancer progression 56. Thus, we considered whether NLRP3 also has an effect on neutrophil polarization. We conclusively proved that CAV1 in BC-derived sEVs promote the neutrophil recruitment through the TLR4-NF-κB-IL-6/CCL2 axis and neutrophil N2-type polarization through the TLR4/NF-κB/NLRP3 pathway.

**Figure 7:**
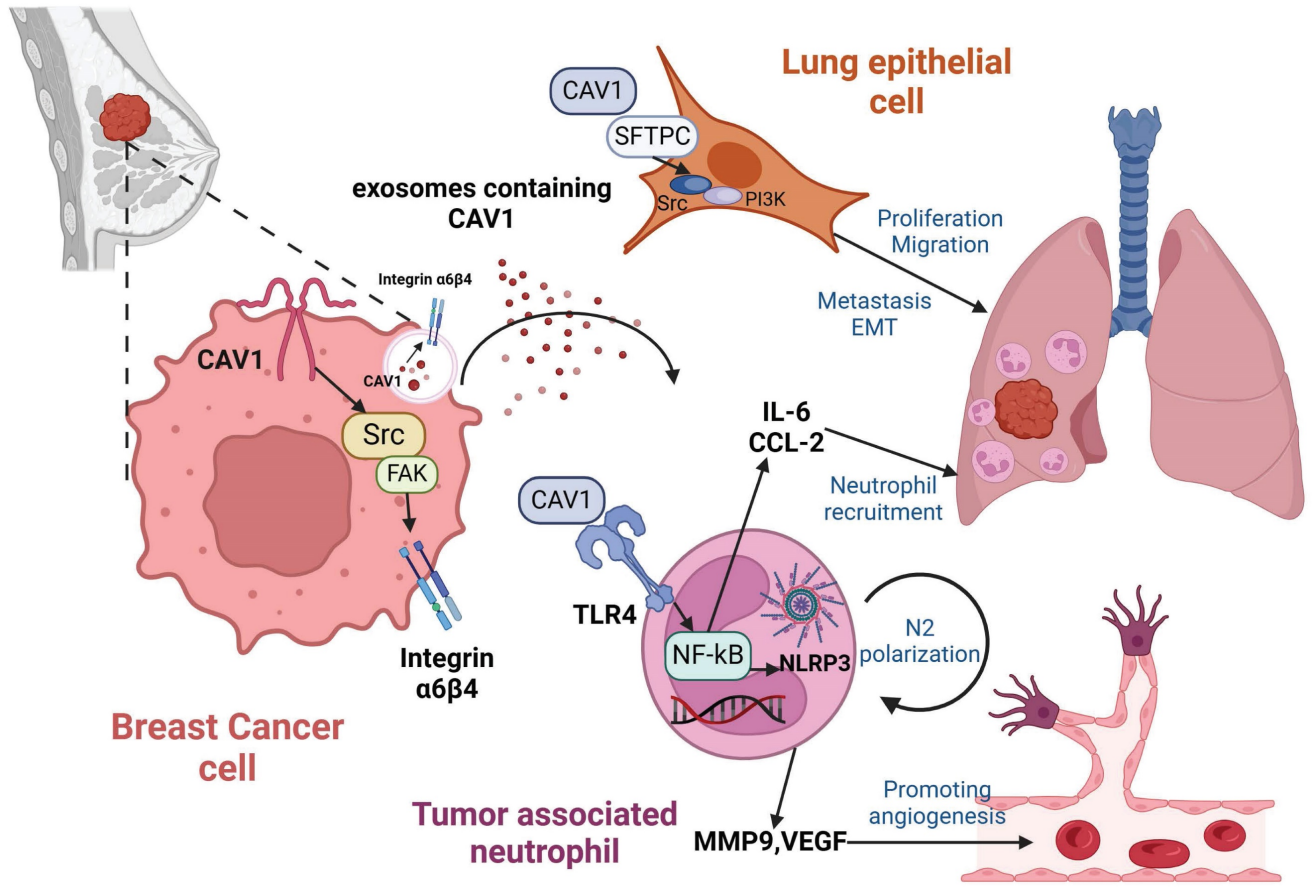
** Mechanisms of BC-derived CAV1 promoting BC lung metastasis.** CAV1 in BC cells promotes integrin α6β4 expression through activation of the Src/FAK pathway, which supports internalization of BC-derived sEVs by lung epithelial cells to enhance lung metastasis, while CAV1 on sEVs promotes neutrophil recruitment in the lung through activation of the TLR4-NF-κB -IL-6/CCL2, and drives N2-type polarization of neutrophils through activation of the TLR4/NF-κB/NLRP3 signaling pathway, and furthermore, CAV1 on sEVs facilitates lung angiogenesis. The figure is created by Biorender.com.

In summary, we found that CAV1 can be transported by BC-derived sEVs. CAV1 alters lung epithelial cell proliferation, invasion, migration capacity and EMT by regulating integrin α6β4 and SFTPC. Additionally, CAV1 promotes neutrophil recruitment through the TLR4-NF-κB-IL-6/CCL2 axis, N2 polarization through the TLR4/NF-κB/NLRP3 pathway and angiogenesis. CAV1 promotes BC lung metastasis through the above pathways (Fig. 7).

## Supplementary Material

Supplementary figures and tables.
