# Supplementary material for: Breast cancer-derived CAV1 promotes lung metastasis by regulating integrin α6β4 and the recruitment and polarization of tumor-associated neutrophils
Source: Int J Biol Sci. 2024 Oct 14;20(14):5695–714. doi: 10.7150/ijbs.94153 (PMC11528463; doi:10.7150/ijbs.94153)
Supplement: Supplementary file 1 — Supplementary figures and tables. [file ijbsv20p5695s1.pdf]

Supplemental Figure S1

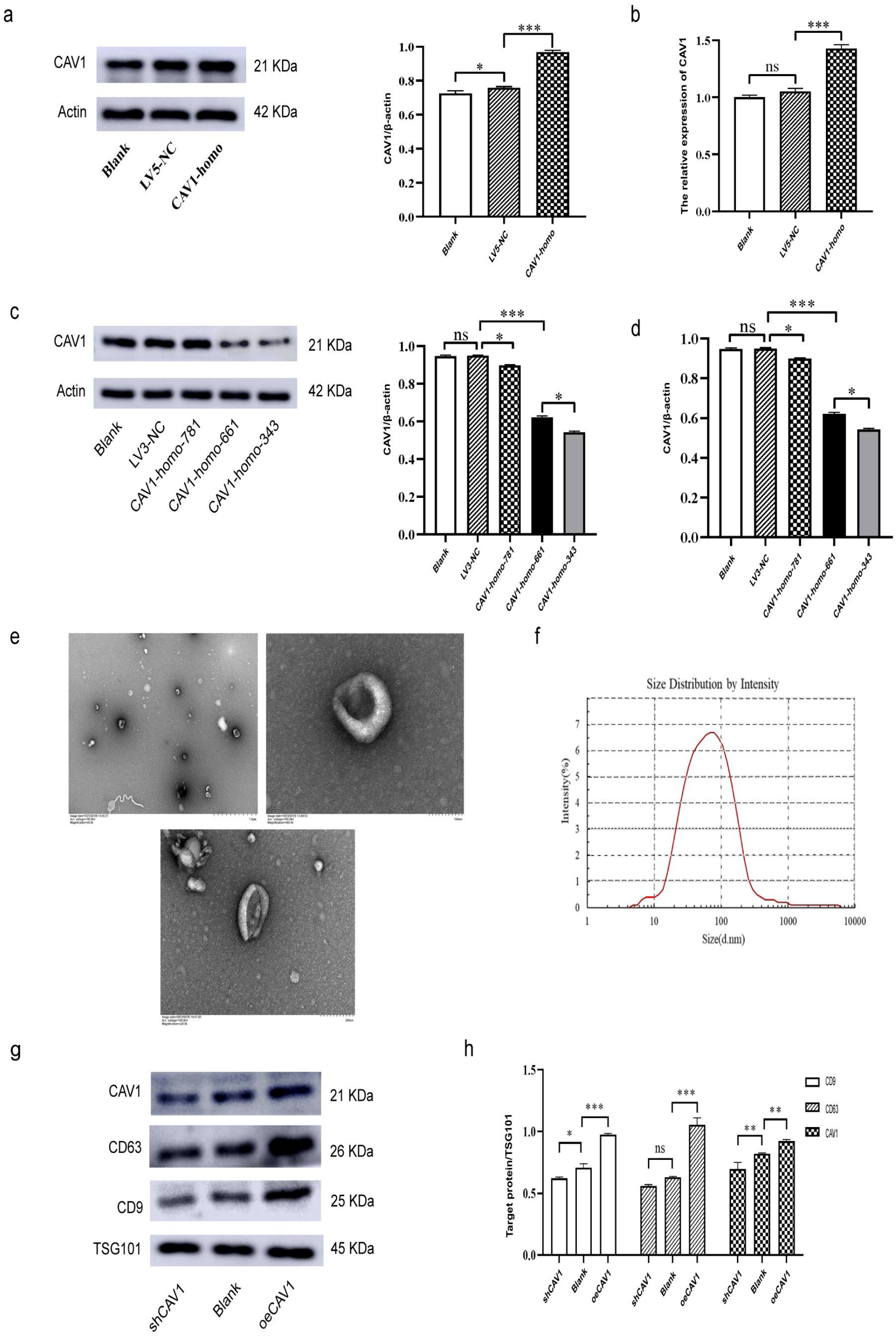

Supplemental Figure S1: A, C: Western blot analysis was performed to detect the expression level of CAV1 in MDA-MB-231 after transfection with different shRNAs. B, D: RT-qPCR detection of CAV1 mRNA levels in MDA-MB-231 after transfection. E: The morphology of sEVs was demonstrated using transmission electron microscopy at various magnifications. The scale for the figures in the upper left, upper right, and lower thirds is respectively 1um, 100nm, and 200nm. F: The results of nanoparticle tracking analyzer showed sEVs diameter. G: Western blot was performed to detect CAV1, EVs markers CD9 and CD63 levels in EVs derived from stably transfected cell lines. H: Statistical analysis of CAV1, CD9 and CD63 protein levels in EVs. Data ware shown as mean ± SD and assessed with One-way ANOVA test. (n=3) (*p<0.05; **p<0.01; ***p<0.001)

Supplemental Figure S2

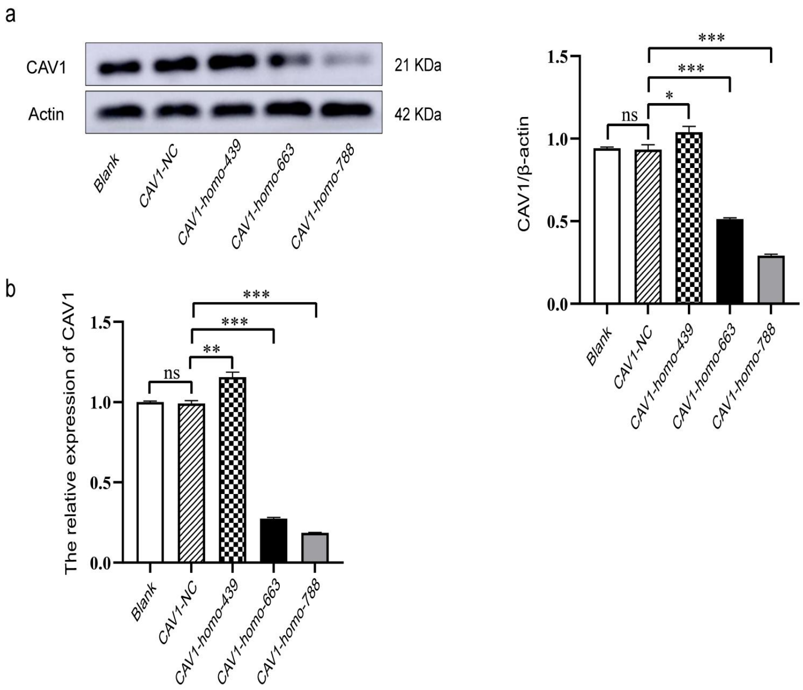

Supplemental Figure S2: A, B: Western blot and RT-qPCR were performed to detect the siRNA fragment that knocked down the CAV1 most significantly in BASE-2B. Data ware shown as mean ± SD and assessed with One-way ANOVA test. (n=3) (ns stands for non-significant difference; *p<0.05; **p<0.01; ***p<0.001)

Supplemental Figure S3

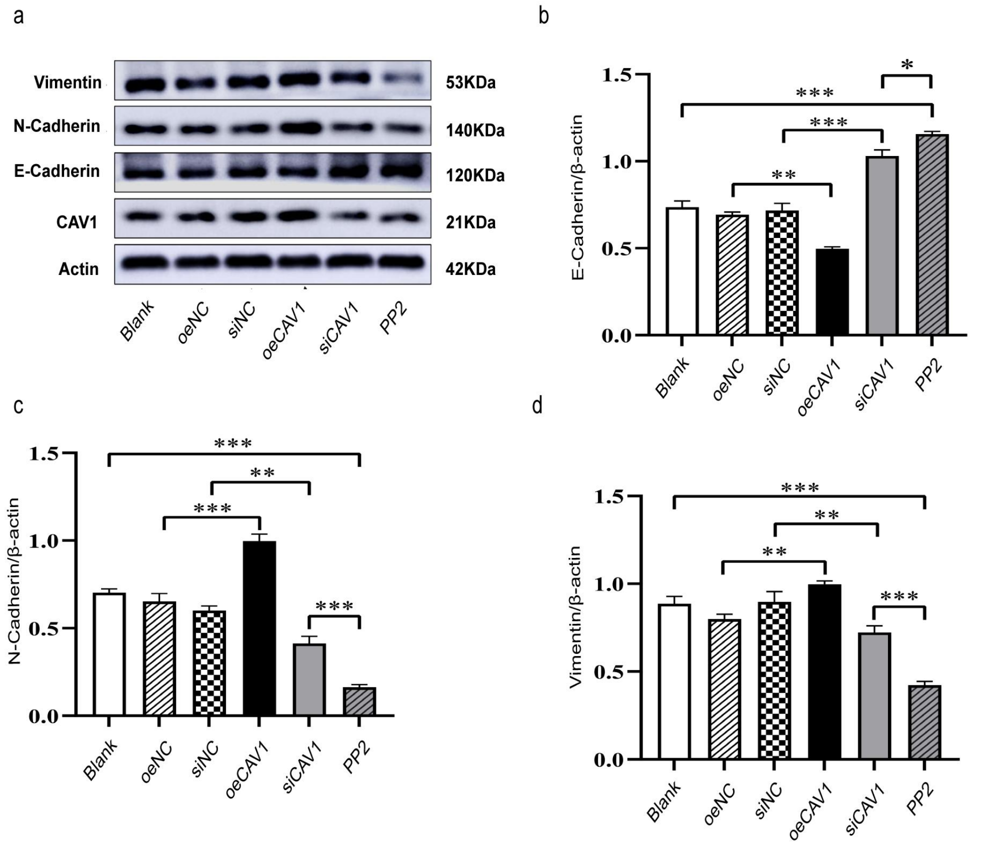

Supplemental Figure S3: A: Western blot was used to detect the expression levels of EMT-related proteins in each group of cells. B-D: Statistical analysis of A. Data ware shown as mean ± SD and assessed with One-way ANOVA test. (n=3) (ns stands for non-significant difference; *p<0.05; **p<0.01; ***p<0.001)

Supplemental Figure S4

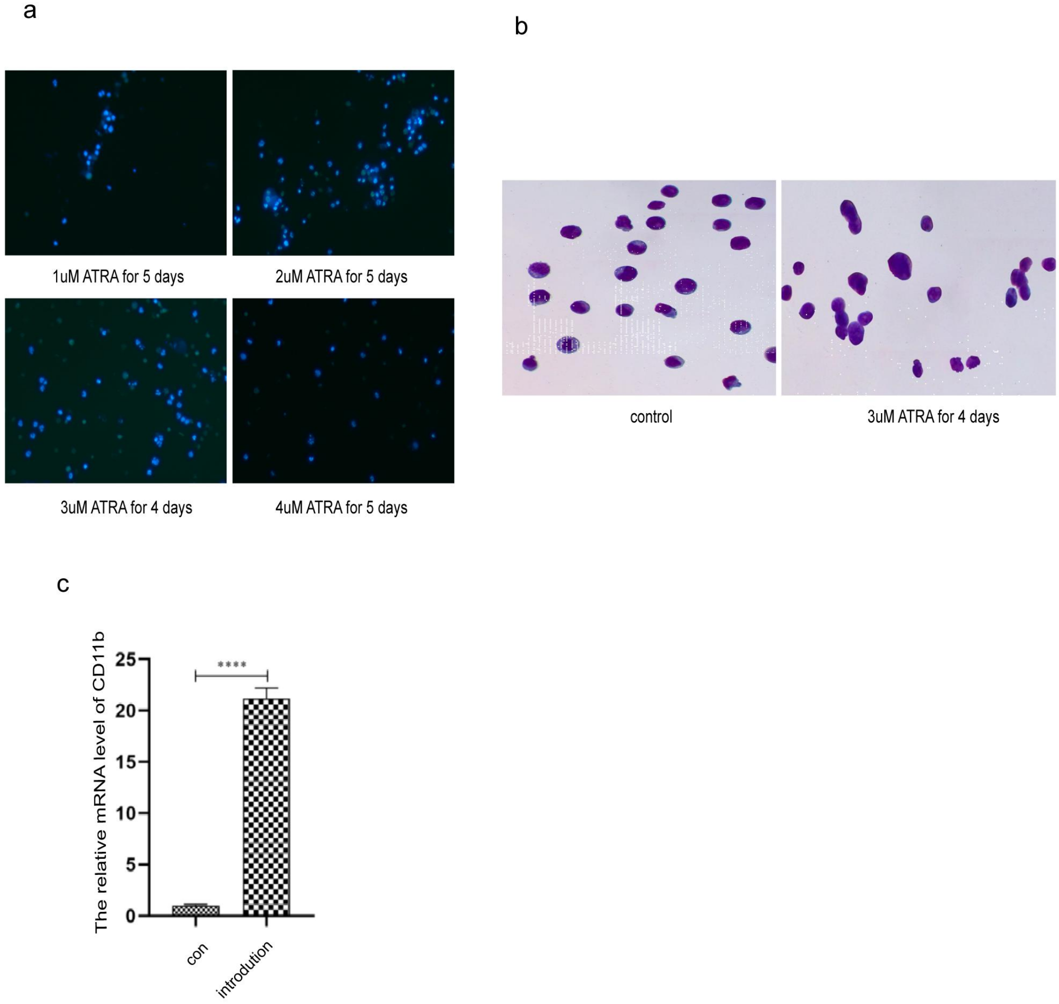

Supplemental Figure S4: Hoechst Stain was used to observe the induction of HL-60 to dHL-60 in each group. Bar=100um. B: Wright-Giemsa Stain was used to observe the number of nuclei in each group. Bar=50um. C: RT-qPCR was used to detect the expression level of dHL-60 marker gene CD11b. Data ware shown as mean ± SD and assessed with One-way ANOVA test. (n=3) (ns stands for non-significant difference; *p<0.05; **p<0.01; ***p<0.001)

Supplemental Table S1: The shRNA nucleotide sequences of LV3-Negative control、LV5-Negative control and Caveolin-1

ID	Sequence (5'to 3'）
LV3-Negative control	TTCTCCGAACGTGTCACGT
LV5-Negative control	NM_001753.5
CAV1-homo-343	GCAACATCTACAAGCCCAACA
CAV1-homo-661	GGGCAGTTGTACCATGCATTA
CAV1-homo-781	GCAATGTCCGCATCAACTTGC
CAV1-homo	NM_001753.5

Supplemental Table S2: The siRNA nucleotide sequences of NC、Caveolin-1 and SFTPC

ID	Sequence (5'to 3'）
Negative control (NC)	UUCUCCGAACGUGUCACGUTT
	ACGUGACACGUUCGGAGAATT
CAV1-homo-439	GCGACCCUAAACACCUCAATT
	UUGAGGUGUUUAGGGUCGCTT
CAV1-homo-663	GCAUGUUGUACCAUGCAUUAT
	UAAUGCAUGGUACAACUGCTT
CAV1-homo-788	CCGCAUCAACUUGCAGAAATT
	UUUCUGCAAGUUGAUGCGGTT
SFTPC-homo-358	GCUGAUCGCCUACAAGCCATT
	UGGCUUGUAGGCGAUCAGCTT
SFTPC-homo-163	CCUCAUCGUCGUGGUGAUUTT
	AAUCACCACGACGAUGAGGTT
SFTPC-homo-492	CCGCAGUGCCUACGUCUAATT
	UUAGACGUAGGCACUGCGGTT
TLR4-homo-1076	CCAGGUGCAUUUAAAGAAATT
	UUUCUUUAAAUGCACCUGGTT
TLR4-homo-1977	CCAGUCUUCAGGUACUAAATT
	UUUAGUACCUGAAGACUGGTT
TLR4-homo-2652	GCUGGUGUAUCUUUGAAUATT
	UAUUCAAAGAUACACCAGCTT

Supplemental Table S3: Sequence of primers used in quantitative RT-PCR

ID	Sequence (5'to 3'）
GAPDH-Forward	CCCACTCCTCCACCTTTGAC
GAPDH-Reverse	CCACCACCCTGTTGCTGTAG
ITGA6-Forward	TCAACAAGGATGGGTGGCAA
ITGA6-Reverse	TCTGCCTTGCTGGTTCATGT
ITGB4-Forward	ATAAGGACTGCGCCTACTGC
ITGB4-Reverse	AAGCTGCTCTCCATGACCAC
CAV1-Forward	GCGACCCTAAACACCTCAAC
CAV1-Reverse	ATGCCGTGTCAAACTGTGTGT
SFTPC-Forward	CTTCTCCATCGGCTCCACTG
SFTPC-Reverse	AGTGAGAGCCTCAAGACTGG
CD11b-Forward	CTTTCTGGCTGAGCTTCGGA
CD11b-Reverse	ACGAGAGGTTGCCTTTGAGG
